# Structure-Function Implications of the Ability of Monoclonal Antibodies Against α-Galactosylceramide-CD1d Complex to Recognize β-Mannosylceramide Presentation by CD1d

**DOI:** 10.3389/fimmu.2019.02355

**Published:** 2019-10-09

**Authors:** Katharine Clark, Jessica Yau, Anja Bloom, Jing Wang, David J. Venzon, Motoshi Suzuki, Lise Pasquet, Benjamin J. Compton, Susanna L. Cardell, Steven A. Porcelli, Gavin F. Painter, Dirk M. Zajonc, Jay A. Berzofsky, Masaki Terabe

**Affiliations:** ^1^Vaccine Branch, Center for Cancer Research, National Cancer Institute, NIH, Bethesda, MD, United States; ^2^Division of Immune Regulation, La Jolla Institute for Allergy and Immunology, La Jolla, CA, United States; ^3^Biostatistics and Data Management Section, Center for Cancer Research, National Cancer Institute, NIH, Bethesda, MD, United States; ^4^Biochemistry and Biophysics Center, National Heart, Lung, and Blood Institute (NHLBI), NIH, Bethesda, MD, United States; ^5^The Ferrier Research Institute, Victoria University of Wellington, Wellington, New Zealand; ^6^Department of Microbiology and Immunology, Institute of Biomedicine, University of Gothenburg, Gothenburg, Sweden; ^7^Department of Microbiology and Immunology and Department of Medicine, Albert Einstein College of Medicine, Bronx, NY, United States; ^8^Department of Internal Medicine, Faculty of Medicine and Health Sciences, Ghent University, Ghent, Belgium; ^9^Neuro-Oncology Branch, Center for Cancer Research, National Cancer Institute, NIH, Bethesda, MD, United States

**Keywords:** natural killer T cells, CD1d, beta-mannosylceramide, alpha-galactosylceramide, L363

## Abstract

iNKT cells are CD1d-restricted T cells recognizing lipid antigens. The prototypic iNKT cell-agonist α-galactosylceramide (α-GalCer) alongside compounds with similar structures induces robust proliferation and cytokine production of iNKT cells and protects against cancer *in vivo*. Monoclonal antibodies (mAbs) that detect CD1d-α-GalCer complexes have provided critical information for understanding of antigen presentation of iNKT cell agonists. Although most iNKT cell agonists with antitumor properties are α-linked glycosphingolipids that can be detected by anti-CD1d-α-GalCer mAbs, β-ManCer, a glycolipid with a β-linkage, induces strong antitumor immunity via mechanisms distinct from those of α-GalCer. In this study, we unexpectedly discovered that anti-CD1d-α-GalCer mAbs directly recognized β-ManCer-CD1d complexes and could inhibit β-ManCer stimulation of iNKT cells. The binding of anti-CD1d-α-GalCer mAb with β-ManCer-CD1d complexes was also confirmed by plasmon resonance and could not be explained by α-anomer contamination. The binding of anti-CD1d-α-GalCer mAb was also observed with CD1d loaded with another β-linked glycosylceramide, β-GalCer (C26:0). Detection with anti-CD1d-α-GalCer mAbs indicates that the interface of the β-ManCer-CD1d complex exposed to the iNKT cell TCR can assume a structure like that of CD1d-α-GalCer, despite its disparate carbohydrate structure. These results suggest that certain β-linked monoglycosylceramides can assume a structural display similar to that of CD1d-α-GalCer and that the data based on anti-CD1d-α-GalCer binding should be interpreted with caution.

## Introduction

**Figure 1:**
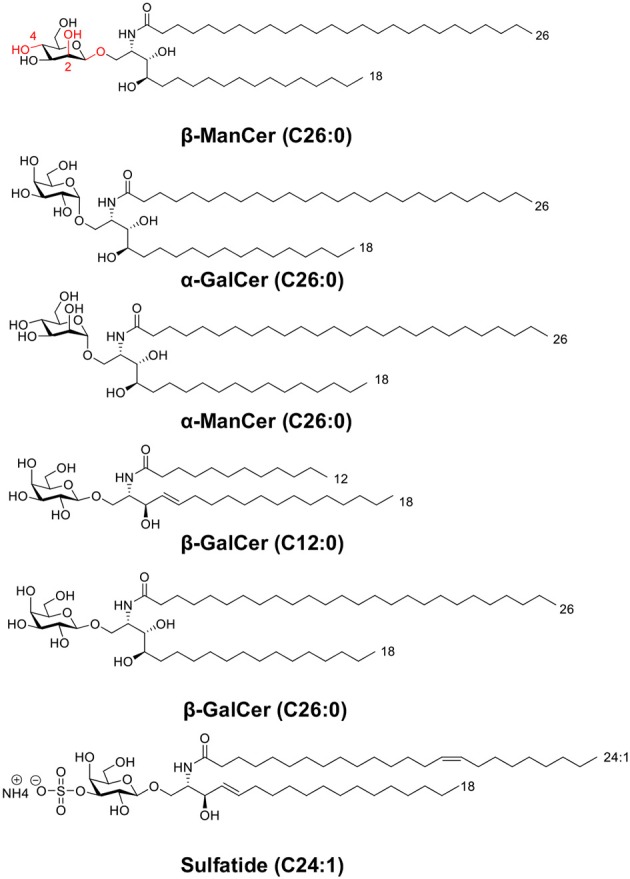
This panel depicts the structures of glycolipids used in this study. The panel includes two sets of anomeric compounds [α-ManCer and β-ManCer, α-GalCer and β-GalCer (C26:0)] as well as β-GalCer (C12:0) and the type II NKT agonist, sulfatide.

Type I natural killer (NK)T cells, or invariant NKT cells (iNKT), express a semi-invariant TCRα Vα14-Jα18 rearrangement paired with a limited Vβ repertoire. Unlike conventional T cells, iNKT cells are restricted by the non-classical major histocompatibility complex (MHC) molecule, CD1d, which presents lipid instead of peptide antigens (1, 2). Upon TCR ligation, iNKT cells rapidly elicit an immune response, producing multiple types of cytokines depending on the stimulus, either directly or through activating downstream effector cells (3–5). The prototypical agonist of all iNKT cells is α-galactosylceramide (α-GalCer, Figure 1). iNKT cells activated by α-GalCer secrete large amounts of cytokines including IFN-γ, IL-4, and IL-13 and undergo proliferation. α-GalCer induces strong anti-tumor immunity *in vivo* through a mechanism that relies on IFN-γ production (6–9). The crystal structure of the iNKT cell TCR-α-GalCer-CD1d complex provides a detailed atomic-level view into the basis of glycolipid binding by CD1d, as well as TCR recognition leading to subsequent iNKT cell activation (10).

α-GalCer is characterized by a ceramide backbone comprised of a C26:0 acyl chain and 18-carbon phytosphingosine chain connected via an α-linkage to a galactose sugar head group (6, 11) (Figure 1). The acyl chain and the phytosphingosine chain of α-GalCer are buried in the hydrophobic A′ and F′ -pockets of the CD1d antigen-binding groove, respectively (12, 13). Consequently, the ceramide structure contributes to α-GalCer's antigenicity at least in part by dictating the ligand's affinity for CD1d. Although the ceramide backbone remains hidden in the cavity of CD1d, the galactose head group is surface-exposed and directly available to contact the iNKT cell TCR and make polar contacts with surface residues on the CD1d molecule (11, 14, 15). The α- or β-linkage of a glycolipid antigen dictates how the glycosyl head protrudes out of CD1d and influences how the iNKT cell TCR recognizes the antigen (16). The iNKT cell TCR adopts a tilted and parallel docking mode over the F′-pocket of CD1d (10). At the interface of the TCR and CD1d-α-GalCer, only the semi-invariant TCRα chain binds to both the glycolipid antigen and CD1d, whereas the TCRβ chain contacts only CD1d residues over the F′ pocket (10). The close interactions between the invariant TCRα chain and galactose head group may account in part for the potency of the antigen in stimulating iNKT cells (11).

Though α-GalCer is the most well-characterized iNKT cell ligand, the iNKT cell TCR binds a diverse assortment of structurally distinct antigens (11) and recognizes several self-glycosphingolipid antigens and β-linked mammalian lipid molecules, such as isoglobotrihexosylceramide (iGb3) and β-galactosylceramide (β-GalCer, Figure 1) (17–19). These β-linked glycosylceramides can activate iNKT cells. For instance, a high dose (50 μg) of β-GalCer induces IFN-γ but not IL-4 in serum after *in vivo* administration in mice, which occurred in an iNKT cell-dependent manner. This glycolipid exacerbates experimental autoimmune encephalomyelitis (EAE), in contrast to the effect of α-GalCer (18). Unlike the more favorable flattened conformation of α-glycosyl head groups, β-linked ligands tend to adopt a perpendicular orientation above the CD1d binding cleft (16, 20, 21). Though seemingly a conundrum, the same iNKT cell TCR is capable of recognizing these disparate glycosphingolipids by flattening β-linked glycolipid antigen-protein complexes upon ligation. This ‘induced-fit molecular mimicry' thereby shapes self β-linked ligands to resemble foreign α-linked antigen structures (21–23). The energetic penalty of converging on this favored footprint may help explain why β-linked ligands are often weaker agonists than are their α-anomer counterparts.

In contrast, another iNKT cell agonist β-mannosylceramide (β-ManCer) exhibits much stronger reactivity than its anomer, α-mannosylceramide (24). Structurally, the β-ManCer used in these studies (Figure 1) is characterized by the same ceramide backbone (C26:0 acyl and C18 phytosphingosine base) as α-GalCer, yet differs significantly in its glycosyl head group, displaying a β-linked mannose sugar rather than an α-linked galactose sugar, and is epimeric at positions 2 and 4 (changes with respect to α-GalCer are marked in red, Figure 1). β-ManCer represents a new class of β-linked antigens capable of inducing potent anti-tumor immune responses largely independent of IFN-γ and completely dependent on NOS and TNF-α and not inducing long-term functional anergy of iNKT cells (24, 25). *In vitro*, β-ManCer is a weaker agonist of iNKT cells than α-GalCer, inducing less cytokine production and a lower expression of activation markers (24). However, following stimulation with either antigen, similar proliferation of iNKT cells with comparable Vβ repertoires can be observed, indicating that β-ManCer stimulates the same subsets of iNKT cells (based on Vβ chain usage) as does α-GalCer.

L363 and L317 are anti-CD1d-α-GalCer mAbs that were developed to specifically bind to CD1d:α-GalCer complexes (26). *In vitro*, unlike anti-CD1d blocking antibodies, they do not recognize unloaded CD1d or CD1d loaded with antigens other than α-GalCer or other α-linked monoglycosylceramides resembling α-GalCer. Anti-CD1d-α-GalCer, L363, showed measurable binding only for CD1d loaded with α-GalCer, α-galactosylphytosphingosine (α-GalPhs) and α-glucosylceramide (α-GluCer), but not for any of the β-linked agonists, including β-GalCer with C24:1 acyl chain and C18 sphingosine base, β-GluCer, or iGb3 at least by the methods used to detect binding (27, 28). The crystal structure of the Fab region of anti-CD1d-α-GalCer bound to CD1d complexed with the α-GalCer analog, C20:2, revealed that anti-CD1d-α-GalCer exhibits iNKT cell TCR-like binding properties, depending on both the heavy and light chains to bind to the antigen-CD1d complex (29). Despite binding similarities between the iNKT cell TCR and anti-CD1d-α-GalCer, the anti-CD1d-α-GalCer antibody L363 does not appear to induce structural changes in the antigen-CD1d complex or reorient the glycolipid head necessary for binding. Thus, L363 cannot recognize the full spectrum of lipid antigens that the iNKT cell TCR can. Instead, an antigen's sugar moiety must be presented nearly identically to α-GalCer to allow for antibody binding. This modality explains why anti-CD1d-α-GalCer has been unable to detect β-linked monoglycosylceramide-CD1d complexes.

Because β-ManCer has the same ceramide structure as α-GalCer, which helps determine the binding kinetics to CD1d, it is likely that β-ManCer interacts with CD1d with comparable affinity as α-GalCer. Yet, it is unknown how β-ManCer is presented by CD1d. Given that iNKT cells can recognize a wide variety of antigens with diverse structures, we exploited the specificity of anti-CD1d-α-GalCer mAbs, which appears stricter than that of the iNKT cell TCR, to investigate structural differences between CD1d-α-GalCer and β-ManCer-CD1d complexes. Surprisingly, we discovered that anti-CD1d-α-GalCer mAbs are capable of detecting CD1d presenting β-ManCer despite its β-linkage. Furthermore, anti-CD1d-α-GalCer mAbs can inhibit the biological activity of β-ManCer to activate iNKT cell hybridoma clones, as well as to activate splenic iNKT cells *ex vivo*. We also discovered that β-GalCer with the same C26:0 ceramide structure with phytosphingosine chain as α-GalCer loaded onto CD1d could be recognized by anti-CD1d-α-GalCer, although the binding was much weaker than that of CD1d-α-GalCer or CD1d-β-ManCer. However, β-GalCer (C12:0), which utilizes sphingosine instead of phytosphingosine and a shorter acyl chain in its ceramide, could not be recognized. Most strikingly, the capacity of anti-CD1d-α-GalCer mAbs to quench iNKT cell reactivity to β-ManCer is nearly equivalent to their inhibition of α-GalCer-induced iNKT cell activation. Importantly, this finding cannot be explained by α-anomer contamination.

To our knowledge, this is the first report of anti-CD1d-α-GalCer mAb recognition of a β-linked glycosylceramide antigen-CD1d complex. Rather than suggesting promiscuity of these antibodies, we found that the monoclonal antibodies' recognition of β-ManCer-CD1d was specific for this particular glycolipid with a β-linkage. These findings indicate that despite having a β-linked sugar head, the specific β-linked glycolipids examined in complex with CD1d, β-ManCer-CD1d and β-GalCer-CD1d, can assume a conformation similar to that of the CD1d-α-GalCer structural complex, allowing it to be captured by anti-CD1d-α-GalCer. This further suggests that the iNKT cell TCR does not need to “force” the β-linked mannose sugar moiety into a favorable conformation to enable binding, helping to explain why β-ManCer exhibits properties unlike other β-linked ligands.

## Methods

### Mice

BALB/c mice were purchased from Animal Production Colonies, Frederick Cancer Research Facility, NCI (Frederick, MD, USA). Animal care was in accordance with the guidelines of the NCI Animal Care and Use Committee. Female mice older than 6 weeks and younger than six months of age were used for all experiments.

### Reagents

The anti-mouse CD1d-α-GalCer antibodies, L363 and L317 (26, 28), were produced by growing antibody-producing hybridoma clones in RPMI-1640 medium (Life Technologies, Frederick, MD) supplemented with 10% ultra-low IgG FCS (HyClone, GE Healthcare, Pittsburgh, PA), with a total of 500 ml in 2-liter roller bottles. The roller bottles were inoculated initially with 2 × 10^8^ hybridoma clones and incubated on a roller bottle apparatus set at 1.6 revolutions/min in a 37°C room in room air environment. After 7 days of incubation, supernatants were harvested, centrifuged to remove debris and cells, and filtered (0.2 micron). IgG was purified using a Protein G Sepharose column (GammaBind, Pharmacia), using 0.5 M acetic acid pH 3.0 for elution. Purified IgG was concentrated to ~2–5 mg/ml and dialyzed extensively against PBS. IgG concentration was determined by optical density at 280 nm. The purified anti-mouse CD1d-α-GalCer antibody, L363, was also purchased from BioLegend, San Diego, CA. The purified anti-CD1d antibody, 20H2, was purchased from Harlan, Indianapolis, IN. mCD1d monomers were obtained from the NIH Tetramer Core Facility, Emory University, Atlanta, GA. CD90.2 magnetic beads were purchased from Miltenyi Biotec, San Diego, CA. Fluorescent protein labeled monoclonal antibodies used in flow cytometry were obtained as follows: Anti-CD1d (clone 1B1) antibody was purchased from BD BioSciences, San Jose, CA. Anti-TCRβ (clone H57-597), anti-CD3 (clone 17A2), anti-CD1d-α-GalCer (clone L363), anti-Ki-67 (clone 16A8), and anti-CD69 (clone H1.2F3) antibodies and avidin-conjugated fluorochromes were purchased from Biolegend, San Diego, CA. PBS57 (α-GalCer analog)-loaded CD1d tetramer was obtained from the NIH Tetramer Core Facility, Emory University, Atlanta, GA. CountBright Absolute Counting Beads were purchased from Invitrogen, Carlsbad, CA. IL-2, IL-4, and IFN-γ ELISA sets were purchased from eBioScience, San Diego, CA.

### Glycolipids

α-GalCer (C26:0) was purchased from Funakoshi, Tokyo, Japan. Sulfatide and β-GalCer (C12:0) were purchased from Avanti Polar Lipids, Alabaster, AL. β-GalCer (26:0) was synthesized as previously described (24).

The synthesis of β-ManCer commenced from the trimethylsilyl trifluoromethanesulfonate promoted addition of 2-azido-3,4-bis-*O*-benzylphytosphingosine to 2,3-bis-*O*-benzyl-4,6-*O*-(phenylmethylene)-D-α-galactopyranosyl trichloroacetimidate generating the protected glycolipid as a mixture of anomers (α:β; 15:85). Reduction of the azide to the amine was mediated by 1M trimethylphosphine in THF, and the beta-anomer was isolated by silica gel chromatography. Acylation with hexacosanoic acid followed by palladium mediated hydrogenolysis of the benzylic protecting groups afforded the target compound β-ManCer as a white solid. NMR and mass spectrometry data were consistent with that previously reported (24). HPLC (Phenomenex Kinetex C18, 2.6 μm, 50 × 3 mm, 40°C, 0.5 mL/min; Mobile phase A = 100:0.1 water/formic acid; Mobile phase B = MeOH; 0–4 min: 60–100% B; 4–12 min: 100% B; 12–13 min: 100–60% B; 13–15 min 60% B) coupled to a Charged Aerosol Detector (CAD) demonstrated a purity of 97.3% for β-ManCer by HPLC-CAD (Figure S1) with the majority of the remaining 2.7% impurities pertaining to methylene homologs which arise from the purity of the starting materials used in the synthesis—namely hexacosanoic acid and phytosphingosine (as evidenced by LCMS, Figure S2). The ^1^H NMR spectrum of β-ManCer (Figures S3, S4) shows that any contaminating α-configured intermediates that arose from the synthetic glycosylation step were purged from the final product. The anomeric proton for α-ManCer- resonates at 4.75 ppm in 2:1 CDCl_3_/CD_3_OD (unpublished data)—a signal which is devoid in the ^1^H NMR spectrum of β-ManCer. Regardless, α-ManCer is a glycolipid that is unable to activate iNKT cells (8, 24) and this study. Thus, any trace α-anomer contamination below the detection limit could not account for the activity.

α-ManCer was synthesized as previously described (24). α-GalCer, β-GalCer, β-ManCer, and α-ManCer were dissolved in 0.5% Tween20 in PBS for *in vitro* use. Sulfatide was dissolved in either 0.5% Tween20 in PBS or DMSO for *in vitro* use.

### Cell Lines

The CD1d-transfected BALB/c 3T3 fibroblast cell line 4D4 (30) was maintained in RPMI 1640 (Life Technologies, Frederick, MD), supplemented with 10% FCS, L-glutamine, sodium pyruvate (1 mM), and non-essential amino acids. The iNKT cell hybridoma clone DN32.D3 was a kind gift from Albert Bendelac (University of Chicago, Chicago, IL). The iNKT cell hybridoma clones 24.9E and 24.8A were generously provided by Samuel Behar (Harvard Medical School, Boston, MA). All iNKT cell hybridoma clones, as well as the type II NKT cell hybridoma clone XV19 (31), were cultured in RPMI 1640 (Life Technologies, Frederick, MD) containing the same supplements listed above, as well as 2-mercaptoethanol (5 × 10^−5^ M).

### Fluorescent Staining of CD1d-Transfectant Cell Line

The BALB/c 3T3 fibroblast cell line 4D4 was pulsed with either vehicle or glycolipids overnight at 37°C. Cells were stained for the presence of CD1d molecules or glycolipid-CD1d complexes on the cell surface with PE-labeled anti-CD1d (1B1, BD BioSciences, San Jose, CA) and/or biotinylated anti-CD1d-α-GalCer (L363) Biolegend, San Diego, CA) followed by avidin-PE (Biolegend, San Diego, CA) antibodies, respectively. The fluorescence of stained cells was measured by FACSCalibur (BD Biosciences, San Jose, CA), and data were analyzed by Flowjo (Tree Star, Ashland, OR).

### iNKT Cell Hybridoma Clone Stimulation Assay

Splenocytes were harvested from mice, and the single cell suspension was depleted of erythrocytes with ACK Lysis Buffer (Lonza, Basel, Switzerland). T-cells were depleted from splenocytes using CD90.2 magnetic beads (Miltenyi Biotec, San Diego, CA) and autoMACS (Miltenyi Biotec, San Diego, CA). The negative fraction was collected and used as a source of antigen presenting cells (APCs). APCs (1 × 10^6^ cells/well) were co-cultured with the hybridoma clone (5 × 10^4^ cells/well) in 96-well round-bottom plate in the presence of exogenous glycolipids or vehicle, with or without 10 μg/ml blocking antibodies (20H2, L363 or L317). After 24-h incubation at 37°C 5% CO_2_, supernatants were collected and IL-2 concentrations were determined by ELISA (eBioScience, San Diego, CA) according to the manufacturer's instructions. Percent inhibition induced by anti-CD1d-α-GalCer antibodies (10 μg/ml) was calculated by comparing IL-2 production after glycolipid stimulation in the absence of antibody (control) to IL-2 production in the presence of antibody using the formula: (1–IL-2_antibody_/IL-2_control_) × 100. In some experiments, plate-bound CD1d was used to stimulate the type II NKT cell hybridoma clone XV19 as follows: mCD1d monomers (NIH Tetramer Core Facility, Emory University, Atlanta, GA) (8 μg/ml) were incubated with vehicle or DMSO-dissolved sulfatide (4 μg/ml) in pH 5 sodium acetate buffer containing saposin C (10 μg/ml) (32) overnight at 37°C. 0.5 μg of mCD1d monomers loaded or not with glycolipid were coated onto 96-well flat bottom plate and incubated overnight at 37°C. The plates were washed with PBS. 5 × 10^4^ cells of the type II NKT cell hybridoma clone XV19 were added to each well in the presence or absence of blocking antibodies, 20H2 or L363 (10 μg/ml). Cells were incubated at 37°C 5% CO_2_ for 24 h. Supernatants were collected and IL-2 concentrations were determined by ELISA (eBioScience, San Diego, CA) according to the manufacturer's instructions.

### *In vitro* iNKT Cell Activation

Splenocytes were harvested from mice, and the single cell suspension was depleted of erythrocytes with ACK Lysis Buffer (Lonza, Basel, Switzerland). Prepared cells (2 × 10^6^ cells/well of 48-well plate) were stimulated for 3 days with vehicle or glycolipid agonist with or without antibodies (20H2, L363 or L317) at 37°C 5% CO_2_. Cells were harvested and the number of iNKT cells was determined by staining with the following reagents: PBS57-CD1d tetramer (NIH Tetramer Core Facility, Emory University, Atlanta, GA) and anti-TCRβ (BioLegend, San Diego, CA) or anti-CD3 (BioLegend, San Diego, CA). Activation of cells was analyzed by staining for intranuclear or surface markers with anti-Ki-67 (BioLegend, San Diego, CA) and anti-CD69 (BioLegend, San Diego, CA) antibodies, respectively. The absolute number of iNKT cells was determined by using CountBright Absolute Counting Beads (Invitrogen, Carlsbad, CA) according to the manufacturer's instructions. The fluorescence of stained cells was measured by FACSCalibur or LSRII (BD Biosciences, San Jose, CA). Data were analyzed by Flowjo (Tree Star, Ashland, OR). Percent inhibition of glycolipid-induced proliferative response induced by anti-CD1d-α-GalCer antibody L317 was calculated by comparing the absolute number of iNKT cells after glycolipid stimulation in the absence of antibody (control) to the absolute number of iNKT cells in the presence of antibody using the formula: (1–iNKT cell #_antibody_/iNKT cell #_control_) × 100.

### *In vitro* Cytokine Assay

Splenocytes were prepared and cultured as previously stated. After 3–4 days of vehicle or glycolipid stimulation, supernatants were collected. The concentration of IFN-γ and IL-4 was determined by ELISA (eBioScience, San Diego, CA) according to the manufacturer's instructions. Percent inhibition of glycolipid-induced cytokine production induced by anti-CD1d-α-GalCer antibody L317 was calculated by comparing IL-4 or IFN-γ production after glycolipid stimulation in the absence of antibody (control) to that in the presence of antibody using the formula: (1–IL-4 or IFN-γ_antibody_/IL-4 or IFN-γ_control_) × 100.

### Surface Plasmon Resonance Studies

Mouse CD1d was expressed in SF9 insect cells and biotinylated as previously reported (29). Aliquots of 5–10 μg of biotinylated CD1d were loaded with a 6-fold molar excess of either porcine brain sulfatides (Avanti Polar Lipids, dissolved in DMSO) or α-GalCer (dissolved in Tween-20 vehicle). Both α-ManCer and β-ManCer were loaded using a 10-fold molar excess in the presence of 0.01 mM Tyloxapol. Loading was performed o/n at RT in 10 μl volumes. As a negative control, recombinant CD1d was incubated in the corresponding buffers, either in the presence (for α- and β-ManCer) or absence (sulfatides, α-GalCer) of 0.01 mM Tyloxapol. Individual CD1d-lipid complexes were immobilized on a CAP sensor chip at response unit levels between 200 and 1,000 (GE Healthcare). Increasing concentrations of L363 IgG (39 pM-5 nM for α-GalCer and β-ManCer and 21.4 to 700 nM for sulfatides and α-ManCer) were passed over the sensor chip for 3 min association and 5 min of dissociation. Kinetic values were obtained in the BiaEval software using the bivalent binding model. Affinity values were obtained by steady state kinetics (RU/conc at ½ of RMax).

### Statistical Analysis

The data were log-transformed where appropriate and analyzed by one-way analysis of variance (ANOVA) or weighted ANOVA, with *p*-values corrected for multiple comparisons by the Hochberg method, using SAS/STAT software version 12.1 (SAS Institute, Cary, NC).

## Results

### β-ManCer and α-GalCer Stimulate iNKT Cell Hybridoma Clones in a CD1d-Dependent Manner

**Figure 2:**
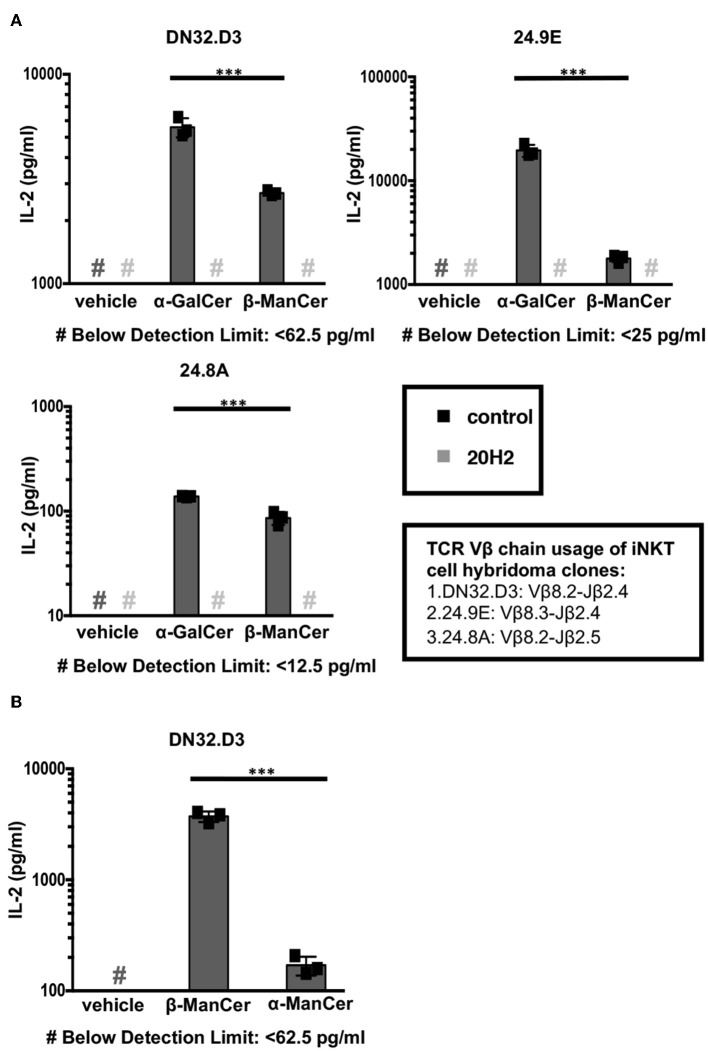
α-ManCer, β-ManCer, and α-GalCer stimulate iNKT cell hybridoma clones in a CD1d-dependent manner. **(A)** The iNKT cell hybridoma clones DN32.D3, 24.9E, and 24.8A were stimulated for 24 h with T cell-depleted splenocytes loaded with vehicle, α-GalCer (100 nM), or β-ManCer (1 μM) in the presence of anti-CD1d antibody 20H2 (10 μg/ml) or in the absence of antibody (control). **(B)** The iNKT cell hybridoma clone DN32.D3 was stimulated with vehicle, β-ManCer (1 μM), or α-ManCer (10 μM). IL-2 concentrations in the supernatant were determined by ELISA **(A,B)**. Data are plotted as mean ± SD of triplicates **(A,B)**. Representative experiments of at least 3 independent repeats **(A)** or 2 independent repeats **(B)** are shown. ***p < 0.0001.

We examined the CD1d-mediated antigen presentation and reactivity to iNKT cells and iNKT cell hybridoma clones of two glycosylceramides, β-ManCer and α-GalCer (Figure 1). iNKT cell hybridoma clones express the invariant Vα14-Jα18 rearrangement paired with different Vβ chains. The amount of IL-2 production corresponds to the strength of iNKT cell TCR signaling in response to the recognition of antigen loaded on CD1d molecules. After overnight stimulation, both β-ManCer and α-GalCer induced a significant amount of IL-2 production relative to vehicle in all three hybridoma cell lines (Figure 2A, Figure S5). The agonistic activity of both glycolipids was determined to be CD1d-dependent, as IL-2 production could be completely abrogated by the addition of the anti-CD1d blocking antibody, 20H2 (Figure 2A). Additionally, β-ManCer stimulated the iNKT cell hybridoma clone DN32.D3 with over 20-fold greater reactivity than its α-anomer, α-ManCer (Figure 2B), which is consistent with previous observations (24). Although the reactivity of glycosphingolipids with β-linkage is often suspected to be due to contamination with α-anomer (28), this finding indicates that the agonistic activity of β-ManCer is due to the β-linked glycolipid itself and cannot be due to contaminants of α-ManCer, because α-ManCer has much weaker reactivity for the iNKT cell TCR.

α-GalCer demonstrated greater reactivity than β-ManCer for all 3 iNKT cell hybridoma clones, which supports our previous data showing that β-ManCer is not as potent of a stimulator of iNKT cells as is α-GalCer (24). However, the order of the magnitude of α-GalCer or β-ManCer stimulation for the different iNKT hybridoma clones expressing different Vβ chains was similar (24.9E ≥ DN32.D3 > 24.8A) (Figure 2A). This finding agrees with the trend observed for the glycolipid-induced proliferation of different Vβ subsets of iNKT cells (24). This preferential stimulation of Vβ chains potentially suggests that at the level of the iNKT cell TCR-Ag-CD1d tripartite complex, the presentation and recognition of β-ManCer may be similar to that of α-GalCer.

### Antibodies Specific for CD1d-α-GalCer Complex Recognize CD1d-Presented β-ManCer

**Figure 3:**
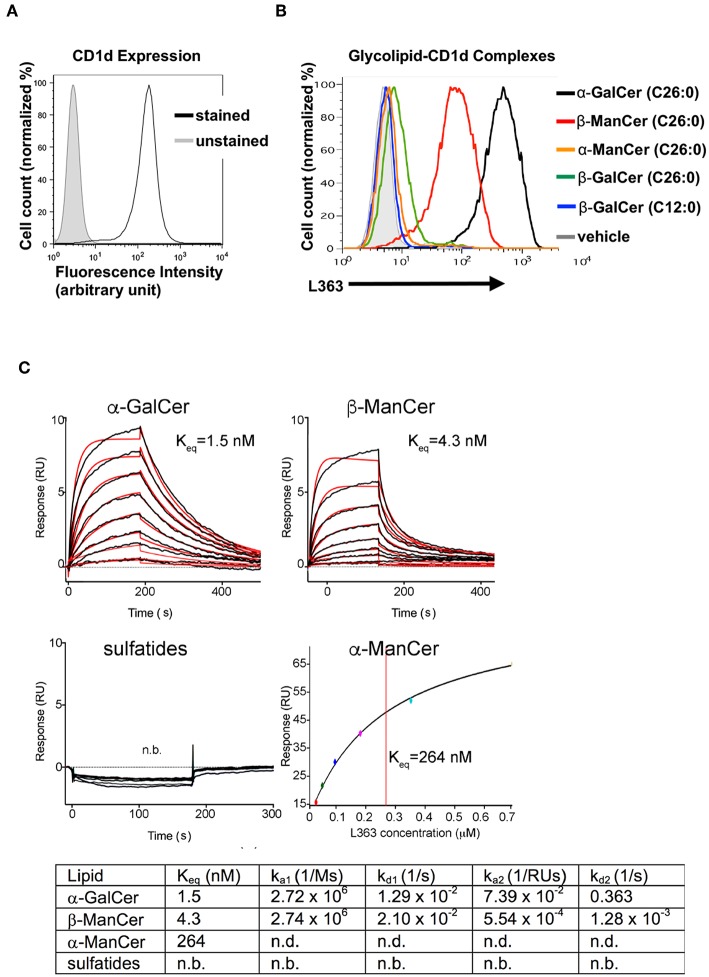
Antibodies specific for CD1d-α-GalCer complexes bind to CD1d pulsed with α-GalCer, β-ManCer and β-GalCer (C26:0), but not β-GalCer (C12:0), α-ManCer, sulfatides or endogenous ligands. **(A)** 4D4 cells were stained or not with anti-CD1d to evaluate the level of expression of surface CD1d molecules. **(B)** 4D4 cells were incubated with vehicle (gray line), α-GalCer 100 nM (black line), β-ManCer 1 μM (red line), α-ManCer 1 μM (orange line), β-GalCer (C26:0) 1 μM (green line) or β-GalCer (C12:0) 1 μM (blue line) overnight. Glycolipid-CD1d complexes were visualized on the cell surface by staining with the biotinylated anti-CD1d-α-GalCer antibody L363 followed by avidin-PE. Representative experiments of 2 independent repeats are shown **(A,B)**. SPR binding analysis of L363 to α-GalCer, α-ManCer, β-ManCer or sulfatides **(C)**. Sensorgrams (black curves) are shown for two-fold dilutions of L363 IgG passed over immobilized CD1d-glycolipid complexes. Bivalent fitted curves (in red) are shown, from which the kinetic data were derived (table). n.b, no binding; n.d., not detected.

L363 and L317 are monoclonal antibodies that were developed against the CD1d-α-GalCer complex. These antibodies have been shown to be able to recognize CD1d loaded with α-GalCer and its analogs, while they do not recognize glycosyl ceramides with a β-linked sugar moiety (iGb3, β-GalCer (24:1), β-psychosine, β-GluCer, β-glucopsychosine) or non-glycosyl ceramides (GalA-GAL, BbGL-2c, Glc-DAG-s2) that can be recognized by the TCR of iNKT cells (26, 28, 29). However, compared to other β-linked glycosyl ceramides, β-ManCer (C26:0) has significantly stronger anti-tumor activity. Thus, we asked whether antibodies specific for the CD1d-α-GalCer complex could detect CD1d-loaded with β-ManCer or rather whether it would discriminate between different ligand-CD1d complexes on the surface of the living cell based on alpha vs. beta linkage. The CD1d-transfectant cell line 4D4 expresses a high level of CD1d (Figure 3A). To test the reactivity of anti-CD1d-α-GalCer for CD1d-β-ManCer complexes, we pulsed 4D4 cells with β-ManCer, α-GalCer, β-GalCer (C26:0 and C12:0) (Figure 1) or vehicle, then stained for antigen-CD1d complexes with the combination of biotinylated anti-CD1d-α-GalCer and avidin-fluorochrome, whose signal was measured by flow cytometry (Figure 3B). In contrast to the cells incubated with vehicle, which demonstrated no measurable binding of anti-CD1d-α-GalCer to CD1d complexes loaded with endogenous lipids, the β-ManCer-pulsed cells unexpectedly could be detected with anti-CD1d-α-GalCer as the α-GalCer-pulsed populations could. However, the level of antibody binding to the α-GalCer-loaded cells was greater than that of β-ManCer-loaded cells. We also pulsed the CD1d-transfectant cells with two other β-linked glycosylceramides, β-GalCer (C26:0) and β-GalCer (C12:0), which have been shown to be recognized by the TCR of iNKT cells (17, 18, 21, 24). β-GalCer (C26:0) is a β-linked anomer of α-GalCer that has the identical ceramide structure as α-GalCer (Figure 1). β-GalCer (C12:0) not only has a shorter acyl chain but also sphingosine instead of phytosphingosine in its ceramide tail. A very weak signal from anti-CD1d-α-GalCer was detected from the cells incubated with β-GalCer (C26:0). In contrast, anti-CD1d-α-GalCer did not bind detectably at all to the cells loaded with β-GalCer (C12:0) (Figure 3B). Finally, consistent with previous observations that the α-anomer of β-ManCer has significantly weaker activity to stimulate iNKT cells, anti-CD1d-α-GalCer failed to recognize CD1d-α-ManCer complexes. This finding indicates that in addition to CD1d-α-GalCer complexes, anti-CD1d-α-GalCer is capable of recognizing CD1d complexes loaded with some, but not all, β-linked glycosylceramides such as β-ManCer or β-GalCer (C26:0). However, the antibody retains its ability to discriminate against some other exogenous or endogenous iNKT cell agonists like β-GalCer (C12:0) and α-ManCer. To our knowledge, this is the first demonstration of anti-CD1d-α-GalCer's binding to a glycolipid with a β-linkage.

### The Antibody Specific for CD1d-α-GalCer Complex Binds β-ManCer With High Affinity

We next asked how the binding affinity of anti-CD1d-α-GalCer antibody, L363, toward CD1d-presented β-ManCer compares to that of α-GalCer, α-ManCer, or sulfatide (another β-linked glycolipid). As expected, sulfatide was not bound by anti-CD1d-α-GalCer, while β-ManCer and α-GalCer were bound with comparable high affinity (Figure 3C). Considering a bivalent binding of the anti-CD1d-α-GalCer to two separate CD1d-ligand molecules on the sensor chip, β-ManCer was bound with an apparent affinity (K_eq_) of 4.3 nM, while α-GalCer was bound with a slightly (half-log) higher affinity of 1.5 nM. While anti-CD1d-α-GalCer appeared to exhibit similar association rates to both glycolipids, the dissociation rate was greater for β-ManCer-CD1d, explaining the slightly reduced binding affinity (Figure 3C). Under the maximal L363 concentration used in the binding assay (5 nM), no binding could be observed toward α-ManCer. However, when we increased the concentration of anti-CD1d-α-GalCer by 120-fold (to 700 nM), we observed binding, albeit of much lower affinity, to α-ManCer (Figure 3C, lower right panel). The association and dissociation rates were too fast to derive kinetic values; however, steady state analysis determined an apparent binding affinity (Kd) of 264 nM, which is roughly 60-fold weaker than that of β-ManCer.

### The Antibodies Specific for the CD1d-α-GalCer Complex Inhibit α-GalCer and β-ManCer Stimulation of iNKT Cell Hybridoma Clones

**Figure 4:**
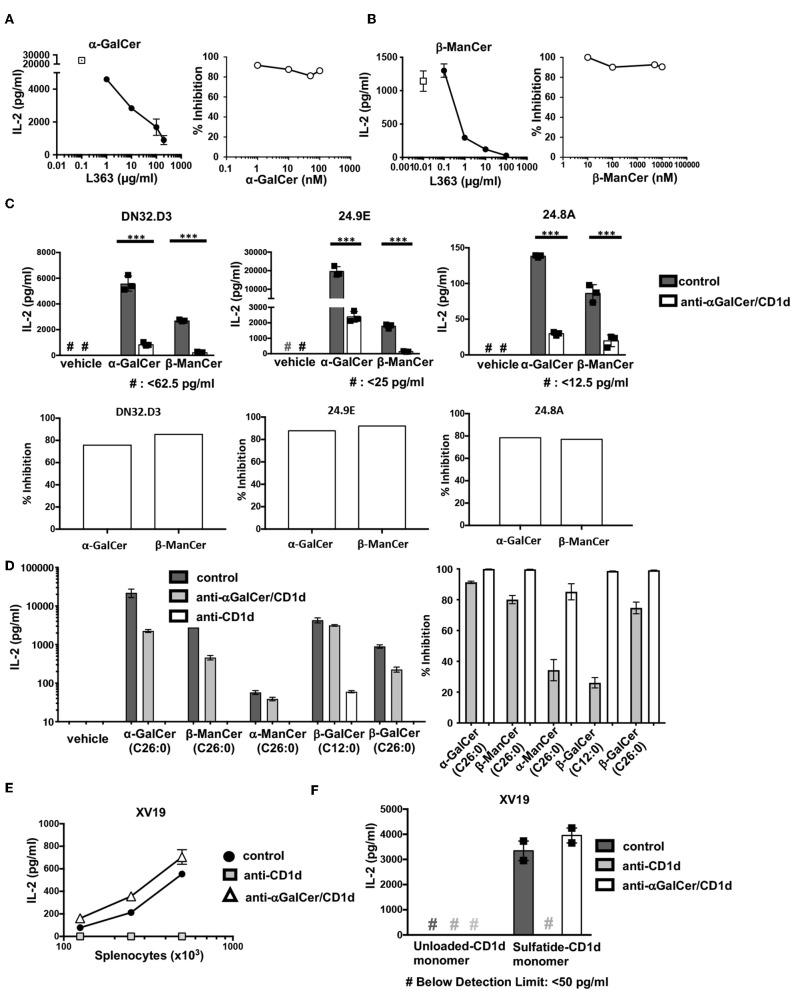
Antibodies specific for CD1d-α-GalCer inhibit α-GalCer- and β-ManCer-induced stimulation of the three iNKT cell hybridoma clones. **(A)** The iNKT cell hybridoma clone DN32.D3 was stimulated for 24 h with T cell-depleted splenocytes loaded with **(A)** 100 nM α-GalCer or **(B)** 1 μM β-ManCer in the presence of increasing concentrations of anti-CD1d-α-GalCer antibody L363 (left panels) or with increasing concentrations of **(A)** α-GalCer or **(B)** β-ManCer in the presence of 10 μg/ml L363 (right panels). Open symbols in left panels indicate IL-2 concentrations without L363. **(C)** The iNKT cell hybridoma clones DN32.D3, 24.9E, and 24.8A were stimulated as described with vehicle, α-GalCer (100 nM), or β-ManCer (1 μM) in the presence of anti-CD1d-α-GalCer antibodies, L363 or L317 (10 μg/ml), or in the absence of antibody (control). Percent inhibition of IL-2 production induced by blocking antibodies was calculated as described in section Methods. **(D)** DN32.D3 was stimulated for 24 h with α-GalCer (100 nM), β-ManCer (1 μM), α-ManCer (1 μM), β-GalCer (C12:0) (1 μM), β-GalCer (26:0) (1μM) in the presence of control IgG, anti-CD1d-α-GalCer or anti-CD1d. **(E)** The type II NKT cell hybridoma clone XV19 was stimulated for 24 h with increasing concentration of T cell-depleted splenocytes in the presence of anti-CD1d (10 μg/ml) or anti-CD1d-α-GalCer (10 μg/ml) or in the absence of antibodies (control). **(F)** XV19 cells were stimulated for 24 h with CD1d monomers (0.5 μg/well) loaded with vehicle or DMSO-dissolved sulfatide in the presence of anti-CD1d or anti-CD1d-α-GalCer (10 μg/ml) or in the absence of antibodies (control). IL-2 concentrations in the supernatant were determined by ELISA. Data are plotted as mean with range of duplicates **(E,F)** or as mean ± SD of triplicates **(A–D)**. Three replicates per experimental group were combined to calculate % inhibition (**A**, right panel; **B**, right panel; **C**, bottom panels; **D**, rights panel). Representative experiments of at least 3 independent repeats **(C)** or 2 independent repeats **(A,B,D–F)** are shown. ***p < 0.0001.

After determining that anti-CD1d-α-GalCer could bind to CD1d-β-ManCer, we tested whether this antibody could inhibit the stimulatory properties of the CD1d-β-ManCer complex. We first characterized the capacity of anti-CD1d-α-GalCer to abolish the reactivity of α-GalCer for the iNKT cell hybridoma clone DN32.D3 to better understand the blocking capabilities of this antibody. Anti-CD1d-α-GalCer reduced α-GalCer-induced stimulation of the iNKT cell hybridoma clone in a dose-dependent manner. When the concentration of α-GalCer was kept constant, a high concentration of anti-CD1d-α-GalCer antibody (100 μg/ml) was needed to induce the greatest amount of inhibition (Figure 4A, left panel). Complete abrogation of α-GalCer-induced stimulation of the iNKT cell hybridoma clone could not be achieved, as residual IL-2 production in the presence of antibody was always observed. When the concentration of α-GalCer was titrated and the concentration of anti-CD1d-α-GalCer was kept constant (10 μg/ml), we observed that the percentage of anti-CD1d-α-GalCer-mediated inhibition showed only minimal dependence on the concentration of α-GalCer used to stimulate the iNKT cell hybridoma clone (Figure 4A, right panel).

After confirming that anti-CD1d-α-GalCer was capable of inhibiting α-GalCer-induced stimulation of the iNKT cell hybridoma clone DN32.D3, we examined the blocking capacity of anti-CD1d-α-GalCer antibody to reduce β-ManCer-induced stimulation of DN32.D3 (Figure 4B). Anti-CD1d-α-GalCer substantially reduced activation of DN32.D3 by β-ManCer in a dose-dependent manner under the conditions in which the concentration of β-ManCer was kept constant. 10 μg/ml of anti-CD1d-α-GalCer inhibited nearly 90% of IL-2 production and almost completely blocked all IL-2 production at a concentration of 100 μg/ml. When the concentration of β-ManCer was titrated with a fixed concentration of anti-CD1d-α-GalCer (10 μg/ml), the magnitude of anti-CD1d-α-GalCer-mediated inhibition showed little or no dependency on the concentration of β-ManCer.

We also examined whether anti-CD1d-α-GalCer could likewise inhibit β-ManCer-induced stimulation of multiple iNKT cell hybridoma clones, and if so, to what extent. Not only did anti-CD1d-α-GalCer inhibit β-ManCer-induced stimulation of all three iNKT cell hybridoma clones (Figure 4C), but also the degree of anti-CD1d-α-GalCer-mediated inhibition of β-ManCer-induced stimulation was comparable to that of α-GalCer-induced stimulation.

Since we observed the binding of anti-CD1d-α-GalCer to the CD1d transfectant, incubated with β-GalCer (C26:0), but not with β-GalCer (C12:0) or α-ManCer (C26:0) (Figure 3B), we tested the ability of anti-CD1d-α-GalCer to inhibit activation of DN32.D3 induced by these glycosphingolipids (Figure 4D). All glycosphingolipids tested activated the iNKT cell hybridoma, although the magnitude of activation measured by IL-2 production was different among them. Consistent with the observation from the binding assay (Figure 3A), anti-CD1d-α-GalCer abrogated the activation induced by α-GalCer, β-ManCer, or β-GalCer (C26:0) while it had a minimal effect on the activation induced by β-GalCer (C12:0) or α-ManCer (C26:0). This finding indicates that anti-CD1d-α-GalCer antibodies recognize CD1d loaded with some glycosphingolipids, which include both α- and β-linked glycosphingolipids, and that upon binding, they block the epitopes required for CD1d-mediated signaling through the iNKT cell TCR. However, these antibodies do not completely mimic the TCR of iNKT cells as they failed to bind CD1d loaded with β-GalCer (C12:0) or α-ManCer (C26:0).

As another control for this inhibition assay, we used the type II NKT cell hybridoma clone XV19, which has demonstrated reactivity to endogenous glycolipids presented by splenocytes (31). Consistent with this previous report, we found that the type II NKT cell hybridoma clone XV19 was autoreactive to splenocytes presenting self-antigens in a dose-dependent manner and that IL-2 production could be abrogated by blocking with anti-CD1d. However, addition of anti-CD1d-α-GalCer to the culture had no effect on the stimulation of the type II NKT cell hybridoma clone XV19, indicating that anti-CD1d-α-GalCer is unable to bind to CD1d-presented endogenous glycolipids that stimulate type II NKT cells (Figure 4E). Blomqvist et al. (31) also demonstrated that the glycosphingolipid sulfatide, an established ligand for type II CD1d-restricted NKT cells, is capable of stimulating the type II NKT cell hybridoma clone XV19. Likewise, we found that sulfatide-loaded CD1d monomers could activate the type II NKT cell hybridoma clone XV19 (Figure 4F). Sulfatide-loaded CD1d monomers induced IL-2 production by XV19, whereas unloaded CD1d failed to induce IL-2 production. Anti-CD1d (20H2) completely abrogated sulfatide-CD1d-induced stimulation, whereas anti-CD1d-α-GalCer had no effect on IL-2 production by the type II NKT cell hybridoma clone XV19. Again, this confirmed that anti-CD1d-α-GalCer did not indiscriminately block CD1d-presented glycolipid induced-activation of the iNKT cell hybridoma.

### The Antibodies Specific for CD1d-α-GalCer Complex Block β-ManCer-Induced Activation of *ex vivo* Splenic iNKT Cells

**Figure 5:**
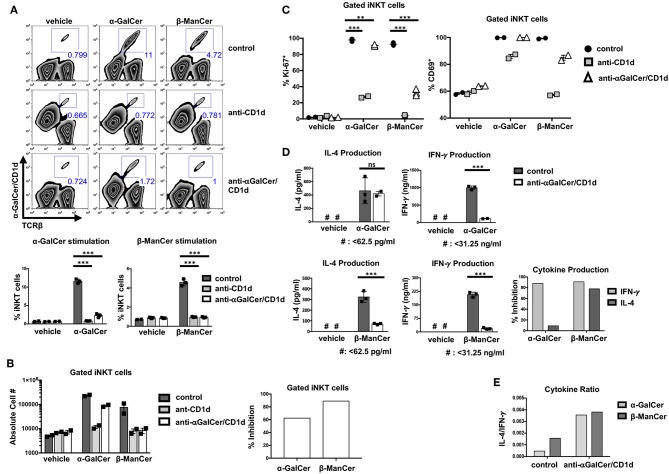
Antibodies specific for CD1d-α-GalCer inhibit glycolipid-induced activation of bulk splenic iNKT cells. **(A)** Mouse splenocytes were stimulated with vehicle, α-GalCer (100 nM) or β-ManCer (1 μM), for 3 days in the presence of anti-CD1d (10 μg/ml) or anti-CD1d-α-GalCer (10 μg/ml for vehicle and β-ManCer; 100 μg/ml for α-GalCer condition) or in the absence of antibodies (control). Proportions of iNKT cells in the culture were enumerated with flow cytometry by using α-TCRβ (or α-CD3) and PBS57/CD1d tetramer. **(B)** The absolute number of iNKT cells was determined by using CountBright Absolute Counting Beads. Percent inhibition of iNKT cell proliferation induced by blocking antibodies was calculated as described in section Methods. **(C)** Mouse splenocytes were stimulated with vehicle, α-GalCer (100 nM), or β-ManCer (1 μM) for 3 days in the presence of anti-CD1d (10 μg/ml) or anti-CD1d-α-GalCer (10 μg/ml for vehicle and β-ManCer conditions; 100 μg/ml for α-GalCer condition) or in the absence of antibodies (control). iNKT cells, gated as in A, were examined for expression of intranuclear Ki-67 or expression of surface CD69. **(D)** Mouse splenocytes were stimulated with vehicle, α-GalCer (100 nM), or β-ManCer (1 μM) for 4 days in the presence of anti-CD1d (10 μg/ml) or anti-CD1d-α-GalCer (10 μg/ml for vehicle and β-ManCer conditions; 100 μg/ml for α-GalCer condition) or in the absence of antibodies (control). Concentrations of IFN-γ and IL-4 in the supernatant were determined by ELISA. Percent inhibition of cytokine production induced by blocking antibodies was calculated as described in section Methods. Data are plotted as mean with range of duplicates **(B,C)** or as mean ± SD of triplicates **(A,D)**. Three replicates per experimental group were combined to calculate % inhibition and cytokine ratio **(B,D,E)**. Representative experiments of 3 independent repeats are shown **(A–E)**. ***p < 0.0001; **p < 0.0040; ns: not significant.

After observing that antibodies specific for anti-CD1d-α-GalCer complex inhibited the glycolipid-induced activation of iNKT cell hybridoma clones, we examined whether these antibodies could prevent the activation of *ex vivo* splenocytes. Splenic iNKT cells were stimulated with α-GalCer, β-ManCer, or vehicle in the presence or absence of the anti-CD1d blocking antibody or in the presence of the antibodies specific for CD1d-α-GalCer complexes. We found that both β-ManCer and α-GalCer induced a significant proliferative response of iNKT cells, as determined by the proportion of iNKT cells after a 3-day culture. α-GalCer always induced a greater proliferative response of iNKT cells than did β-ManCer (Figure 5A). Addition of anti-CD1d antibody abrogated *in vitro* β-ManCer- and α-GalCer-stimulation of iNKT cells, such that the proportion of iNKT cells remained at approximately 1% after three days. This confirmed the CD1d-dependent presentation of these antigens in this setting. In anti-CD1d treated samples, TCRβ^−^ cells were weakly stained with PBS57-loaded CD1d-tetramers (Figure 5A, second row). This may be caused by binding of anti-CD1d antibodies to the surface of TCRβ^−^ cells and then subsequent capture of CD1d- tetramers by these antibodies. Similar to the blocking effects achieved by anti-CD1d, anti-CD1d-α-GalCer reduced the magnitude of both the β-ManCer- and α-GalCer-induced proliferative response of iNKT cells.

Not only did anti-CD1d-α-GalCer with specificity for CD1d-α-GalCer complexes diminish the proportion of iNKT cells after stimulation with either α-GalCer or β-ManCer (shown for anti-CD1d-α-GalCer in Figure 5A, third row), but also it prevented an increase in the overall absolute number of iNKT cells in the culture (shown for anti-CD1d-α-GalCer in Figure 5B). The magnitude of inhibition induced by anti-CD1d-α-GalCer appeared to be similar for both the α-GalCer and β-ManCer responses (Figure 5B). However, 100 μg/ml (or greater) concentration of anti-CD1d-α-GalCer was necessary to achieve a comparable inhibitory effect for the α-GalCer response to what 10 μg/ml of antibody could achieve for the β-ManCer response (data not shown). Because the stimulatory capacity of CD1d-α-GalCer is greater than that of CD1d-β-ManCer, it is possible that fewer free CD1d-α-GalCer complexes are needed to sufficiently activate iNKT cells. Thus, 10 μg/ml anti-CD1d-α-GalCer is unable to fully compete and quench the reactivity of α-GalCer, and a higher concentration of antibody is needed to achieve an equivalent effect.

As another measure of proliferation, we examined the upregulation of the cell cycle marker, Ki-67, after glycolipid stimulation. Although α-GalCer induced a more robust proliferative response after a 3-day stimulation, most iNKT cells became Ki-67^+^ after stimulation with either α-GalCer or β-ManCer (Figure 5C, left panel). In contrast, a minimal level of Ki-67 expression was detected in iNKT cells that were stimulated with vehicle alone, which indicates the null baseline expression of this cell cycle marker (Figure 5C). While anti-CD1d significantly abrogated α-GalCer- or β-ManCer-induced upregulation of Ki-67 in iNKT cells, anti-CD1d-α-GalCer could only partially inhibit Ki-67 upregulation after β-ManCer stimulation. For the α-GalCer response, anti-CD1d-α-GalCer only marginally reduced Ki-67 upregulation in iNKT cells (in terms of magnitude of reduction), though the observed difference was statistically significant (Figure 5C). This finding again likely reflects the inability of the antibody to completely saturate all the sites, and the ability of even a low density of unblocked CD1d-α-GalCer complexes to trigger iNKT cell activation. Although Ki-67 is not expressed in resting cells, this marker appears after antigen stimulation in the late G1 phase of the cell cycle, and expression remains elevated throughout the remainder of mitosis (33). Considering the function and temporal expression of Ki-67, it appears that β-ManCer prepares most NKT cells for a proliferative response, even if that response is more marginal than the one induced by α-GalCer. Furthermore, although the upregulation of Ki-67 in iNKT cells may be an indicator of cells entering the cell cycle, it does not always predict the magnitude of subsequent proliferation.

As yet another marker of activation, we examined changes in CD69 expression after glycolipid-induced iNKT cell stimulation (Figure 5C, right panel). The activation marker CD69 is expressed on at least 50% of splenic iNKT cells at baseline and can be upregulated by stimulating iNKT cells with α-GalCer or β-ManCer, whereby approximately 100% of iNKT cells become CD69^+^ (Figure 5C). Blocking either α-GalCer or β-ManCer activation with anti-CD1d decreased the upregulation of CD69, particularly for the β-ManCer response. Anti-CD1d-α-GalCer, in contrast, had no effect on α-GalCer-induced CD69 upregulation on the surface of iNKT cells and only a marginal effect on β-ManCer-induced CD69 upregulation. This finding is somewhat discrepant with the partial effect anti-CD1d-α-GalCer has on the inhibition of Ki-67 for the β-ManCer response (Figure 5C) or for cell expansion (Figure 5A), but may reflect a lower threshold for upregulation of the CD69 activation marker than for induction of cell cycling.

It is well-known that different iNKT glycolipid agonists can induce different cytokine profiles. We evaluated β-ManCer- and α-GalCer-induced activation of splenic iNKT cells by measuring the level of cytokines in the supernatant after a 4-day stimulation. Though both α-GalCer and β-ManCer induced production of IFN-γ and IL-4, α-GalCer induced greater IFN-γ and IL-4 production than did β-ManCer (Figure 5D). The difference in the potency of α-GalCer and β-ManCer in the induction of various cytokines is consistent with past observations demonstrating that β-ManCer induces low levels of IFN-γ, IL-4, IL-13, and TNF-α (24). Interestingly, β-ManCer-stimulated spleen cells produced a higher ratio of IL-4 to IFN-γ than did α-GalCer-stimulated cells, thereby skewing the cytokine profile slightly toward more IL-4 production (Figure 5E). The addition of anti-CD1d-α-GalCer greatly reduced the level of β-ManCer-induced IFN-γ and IL-4 production, whereas for α-GalCer, anti-CD1d-α-GalCer could inhibit only IFN-γ but not IL-4 production. Thus, the magnitude of anti-CD1d-α-GalCer-induced inhibition of IFN-γ production was comparable for the β-ManCer and α-GalCer conditions, but significantly different between agonists for IL-4 production (Figure 5D). Anti-CD1d-α-GalCer's partial cytokine blockade after α-GalCer stimulation compared to the more robust cytokine blockade after β-ManCer stimulation not only increased the IL-4/ IFN-γ ratio for both agonists but also equalized the ratio of IL-4 to IFN-γ between the two agonists (Figure 5E). The pathway of IL-4 production can be induced by a weaker TCR-ligand interaction than is required for IFN-γ production (34). If TCR signaling is disrupted before the IFN-γ machinery is turned on, cells can still produce IL-4 with very limited production of IFN-γ. Therefore, it is reasonable that anti-CD1d-α-GalCer mitigates IFN-γ production yet remains unable to influence α-GalCer-induced IL-4 production. This observation aligns with the notion that the anti-CD1d-α-GalCer antibodies seem unable to completely block the reactivity of CD1d-α-GalCer complexes to stimulate iNKT cells. The cytokine data are therefore consistent with the limited or lack of inhibitory effect anti-CD1d-α-GalCer has on the markers of activation, Ki-67 and CD69, and the increased concentration of antibody needed to abrogate the α-GalCer-induced proliferative response of iNKT cells.

### α-GalCer Is a More Potent Agonist Than β-ManCer, Inducing Activation of iNKT Cells at Lower Concentrations

**Figure 6:**
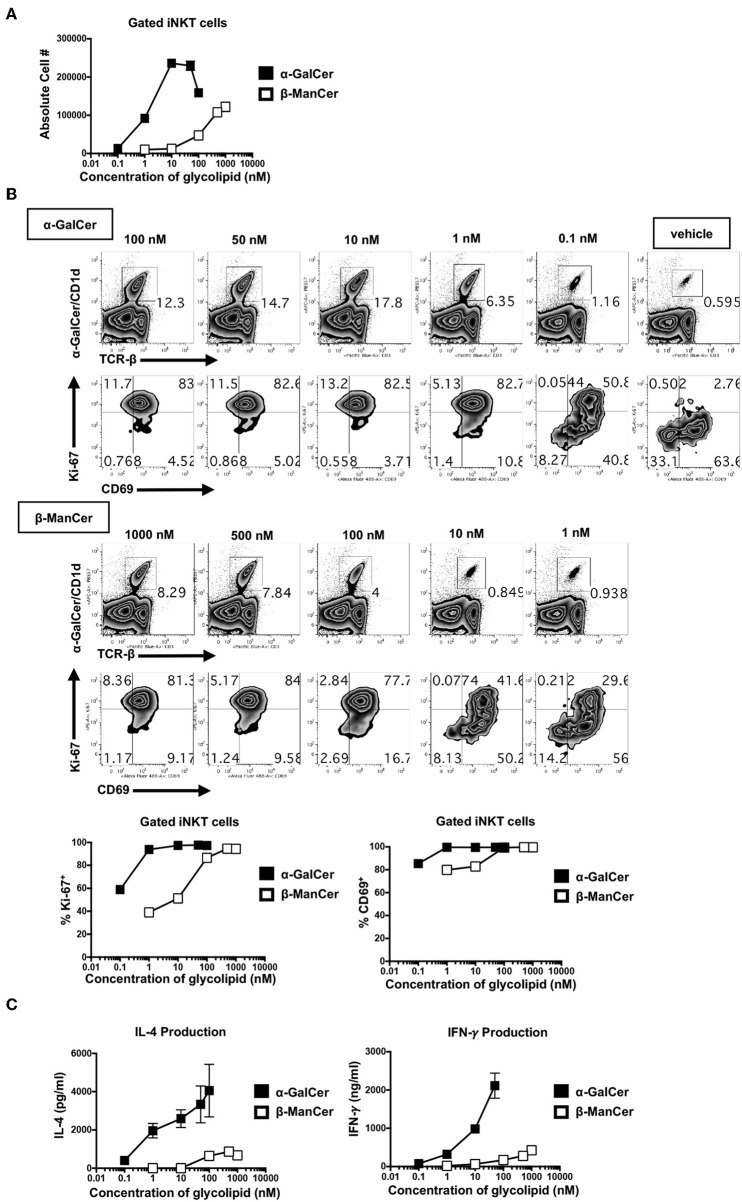
α-GalCer is 100-fold more potent than β-ManCer, inducing Ki-67 and CD69 upregulation and increased cytokine production at lower stimulatory concentrations. **(A)** Mouse splenocytes were stimulated with vehicle or decreasing concentrations of serially-diluted α-GalCer or β-ManCer for 3 days. iNKT cells were identified as TCRβ (or CD3)^int^PBS57-CD1d tetramer^+^ using flow cytometry and were enumerated with CountBright Absolute Counting Beads. **(B)** iNKT cells, gated as in A, were examined for intranuclear upregulation of Ki-67 or expression of CD69 on the cell surface. **(C)** Concentrations of IFN-γ and IL-4 in the supernatant were determined by ELISA. Data are plotted as mean ± range of duplicates **(A–C)**. Representative experiments of 2 independent repeats are shown **(A–C)**.

To better dissect the differences in the inhibitory activity of anti-CD1d-α-GalCer for β-ManCer- and α-GalCer-induced iNKT cell activation, the relative stimulatory properties of each antigen was examined. Concurrent serial dilutions of both agonists revealed that α-GalCer was at least 100-fold more potent than β-ManCer (Figure 6A). In terms of absolute cell number in the culture, α-GalCer induced a higher peak of iNKT cell proliferation (Figure 6A). Anti-CD1d-α-GalCer partially inhibited β-ManCer-, and only slightly inhibited α-GalCer-, induced upregulation of Ki-67, but had little to no effect on either β-ManCer- or α-GalCer-induced CD69 expression (Figure 5C). By decreasing the stimulatory concentration of both agonists, we found that iNKT cell Ki-67 expression declined more precipitously in the β-ManCer response than in the α-GalCer response (Figure 6B). In contrast, CD69 expression remained upregulated even at low concentrations of either antigen. Indeed, the loss of Ki-67 seemed to precede the loss of CD69 in the titration, so that largely the only cells that had lost CD69 expression had also lost Ki67 expression. Thus, fewer free glycolipid-CD1d complexes are necessary for activation of iNKT cells measured by surface CD69 expression, while stronger antigenic stimulation is necessary to induce changes in the cell cycle.

Similarly, though α-GalCer induced more copious cytokine production (IFN-γ and IL-4) than did β-ManCer at every concentration of glycolipid measured, the overall amount of IFN-γ produced was more sensitive to decreasing concentrations of antigen than was IL-4 (Figure 6C). This likely explains why anti-CD1d-α-GalCer successfully diminished α-GalCer-mediated IFN-γ production, while having a negligible effect on IL-4 production, because the number of available lipid-CD1d complexes that must be blocked to inhibit IL-4 production is greater than to inhibit IFN-γ production. This finding indicates that in order to induce a more robust IFN-γ response, the frequency of CD1d-α-GalCer complexes should be maximized. Interestingly, these antibodies specific for CD1d-α-GalCer complexes may provide a tool by which to modify the IL-4/IFN-γ cytokine balance, as well as to possibly alter the production of other cytokines in the milieu, in response to glycolipid stimulation of iNKT cells (Figure 5E).

## Discussion

Given the consistent capability of monoclonal antibodies specific for CD1d-α-GalCer complex (L363 and L317) to inhibit β-ManCer-stimulation of iNKT cells in a variety of biological assays, the direct measurement of binding affinity of anti-CD1d-α-GalCer to CD1d-β-ManCer complex by plasmon resonance, and flow cytometry, we concluded that these monoclonal antibodies, which were developed to be specifically reactive to the CD1d-α-GalCer complex, also recognize and functionally bind to the CD1d-β-ManCer complex. Unlike the iNKT cell TCR, these anti-CD1d-α-GalCer antibodies were reported to be unable to induce structural changes in both antigen and CD1d to recognize disparate lipid antigens (21, 29). Because anti-CD1d-α-GalCer fails to reorient the glycolipid head necessary for binding, it is likely that the complex that β-ManCer forms with CD1d is structurally analogous to CD1d-α-GalCer at least part of the time. Otherwise, anti-CD1d-α-GalCer would be unable to capture CD1d-presented β-ManCer. This finding is particularly intriguing considering the nature of the β-linked mannose sugar moiety of β-ManCer relative to the α-linked galactose head group of α-GalCer.

The binding of anti-CD1d-α-GalCer to β-linked glycosylceramide was not unique to β-ManCer since it could also bind to β-GalCer (C26:0). However, it did not bind to either α-ManCer or β-GalCer (C12:0). It was quite surprising that the two β-GalCer variants tested, β-GalCer (C26:0) and β-GalCer (C12:0), provided completely opposing results in terms of binding to the anti-CD1d-α-GalCer. These two β-GalCer can be distinguished by two structural differences (Figure 1). The first difference is the length of the acyl chain, while the second is the incorporation of either a phytosphingosine in the C26:0 version or a sphingosine chain in the C12:0 version. More importantly, β-GalCer (C12:0) had stronger biological activity to stimulate the iNKT cell hybridoma. Thus, β-GalCer's (C12:0) failure to bind with anti-CD1d-α-GalCer was not because of its assuming a structure that could not be recognized by iNKT cell TCRs. With the anti-CD1d-α-GalCer's inability to force an induced fit (26, 28, 29), it is likely that β-GalCer (C12:0) loaded with CD1d has a structure that is significantly different from that of CD1d-α-GalCer complex, yet one that retains the ability to be recognized by the iNKT cell TCRs. To this end, it is important to note that the β-GalCer (C26:0) compound shares both the C26:0 acyl chain and the phytosphingosine chain with the prototype ligand α-GalCer, and so differs only in the beta vs alpha linkage, whereas the C12:0 version differs in all three structural features.

Anti-CD1d-α-GalCer could stain CD1d loaded with β-ManCer or β-GalCer (C26:0) on the surface of the living cell, although the amount of signal detected by flow cytometry was approximately 1 log and 2.5 log lower than for the α-GalCer-pulsed population, respectively. The signal from anti-CD1d-α-GalCer bound with β-GalCer (C26:0)-loaded CD1d was detected only when a biotinylated anti-CD1d-α-GalCer was combined with an avidin-labeled fluorochrome, but not with the antibody directly labeled with a fluorochrome. Both β-ManCer and β-GalCer (C26:0) have the identical ceramide structure as α-GalCer. The weaker signal of anti-CD1d-α-GalCer bound to the β-ManCer-loaded or β-GalCer (C26:0)-loaded CD1d molecules is likely attributable to either a lower binding affinity of anti-CD1d-α-GalCer to the antigen-CD1d complex, or a lower loading efficiency of β-ManCer and β-GalCer (C26:0) to CD1d. The one-log difference in the signal levels of anti-CD1d-α-GalCer to β-ManCer, compared to α-GalCer, is more than can be explained by the half-log (3-fold) lower affinity of the antibody to the antigen CD1d complex measured by plasmon resonance (Figure 3C). Thus, only some of the difference in staining may be due to lower affinity of β-ManCer for CD1d, which is interesting considering that β-ManCer has the same ceramide structure as α-GalCer. If β-ManCer has a lower CD1d binding efficiency than α-GalCer, then fewer β-ManCer-CD1d molecules would be available for anti-CD1d-α-GalCer to capture, leading to lower signal detection (although we compensate in part by using a higher concentration of β-ManCer).

On the other hand, anti-CD1d-α-GalCer's weaker recognition of β-ManCer-CD1d than that of CD1d-α-GalCer could be explained by two different antigen-presenting modalities. In one modality, α-GalCer and β-ManCer assume fixed structures protruding out of CD1d. The off rates of anti-CD1d-α-GalCer for these rigid antigen-CD1d complexes would then determine the relative binding affinities that were observed via flow cytometry. In the second modality, one we consider more plausible, β-ManCer assumes various conformations in the complex with CD1d, oscillating between a conformation that resembles CD1d-α-GalCer and other conformations that assume very different, unknown, and potentially weaker agonistic displays. Such “breathing” or conformation fluctuation is typical for proteins studied in solution. The lower agonistic activity of β-ManCer for iNKT cells could be due to the small fraction of molecules present in the optimal conformation at any one time. In this scenario, because anti-CD1d-α-GalCer specifically recognizes CD1d-α-GalCer, anti-CD1d-α-GalCer can bind only β-ManCer-CD1d molecules that assume the favorable conformation. The conformations may be present a minority of the time but with enough frequency to enable anti-CD1d-α-GalCer recognition. The amount of time β-ManCer-CD1d spends in the relevant conformation accounts for the lower binding affinity anti-CD1d-α-GalCer has for the β-ManCer-CD1d complex, as fewer β-ManCer-CD1d molecules are present as the complex structure that can be recognized by the monoclonal antibody. Once the antibody binds a CD1d-β-ManCer complex, it locks in the conformation that binds, and pulls the conformational equilibrium toward the favorable conformation until most or all the complexes are in that conformation. The half-log difference in apparent affinity of anti-CD1d-α-GalCer for β-ManCer-CD1d vs. CD1d-α-GalCer suggests that only about 1/3 of the free β-ManCer-CD1d molecules (not bound to the antibody) assume the right conformation at any one time.

In the future, we hope that crystallography will resolve the structure of β-ManCer-CD1d- anti-CD1d-α-GalCer, thereby elucidating the nature of the anti-CD1d-α-GalCer interaction with the antigen-CD1d complex and perhaps lending credence to one antigen-presenting modality or the other. Comparing this structure to the β-ManCer-CD1d-iNKT cell TCR tripartite complex (which also remains unresolved), as well as to the well-characterized structures of CD1d-α-GalCer- anti-CD1d-α-GalCer and α-GalCer-CD1d-iNKT cell TCR (11, 29, 35), will illuminate the differential chemical bonds that β-ManCer forms with the iNKT cell TCR.

Discovering that antibodies reactive to α-linked monoglycosylceramide-CD1d complexes also react to β-ManCer-CD1d has added another layer of complexity to our understanding of this novel iNKT cell agonist. Superficially, β-ManCer, like α-GalCer, activates iNKT cells and induces strong anti-tumor immunity *in vivo*. However, unlike α-GalCer, which depends on IFN-γ production to achieve protection, the protection induced by β-ManCer relies entirely on NOS and TNF-α and not IFN-γ (24). Whereas, α-GalCer induces long-term functional anergy of iNKT cells, β-ManCer does not (25). Despite evoking different pathways of protection, both α-GalCer and β-ManCer act on the same effector cell population, and based on the current study, they appear to be presented by CD1d in an analogous manner.

How β-ManCer can be presented like α-GalCer by CD1d, transducing signals through the same iNKT cell TCR yet inducing diverse effector functions, is outwardly puzzling. However, it is well-known that analogs of α-GalCer that have modifications in the head group or acyl or sphingosine chains bias downstream immune responses through differential cytokine production (11, 36). Even though these analogs closely resemble α-GalCer and can be recognized by anti-CD1d-α-GalCer (28, 29), subtle alterations within the iNKT cell TCR-antigen-CD1d interface undoubtedly influence iNKT cell function (37). This could be analogous to altered peptide ligands for conventional T cells that can induce different activities such as cytokine profiles (38–40). Considering furthermore that CD1d-presented β-ManCer may oscillate between favorable and unfavorable conformations, it is not surprising that β-ManCer differs from α-GalCer in its ability to affect downstream pathways.

Interestingly, this finding might also have important ramifications for the recent debate that α-GalCer can be a potential endogenous ligand for iNKT cells (27, 28). Anti-CD1d-α-GalCer was used to identity the location of endogenous ligands in tissues in the study. However, as we showed in this study, this antibody is capable of binding to CD1d loaded with certain species of β-GalCer or other β-linked glycosylceramides, the interpretation of some data based on anti-CD1d-α-GalCer may need to be done with caution. The structural characteristics of β-linked glycosylceramides, especially endogenous ones recognized by anti-CD1d-α-GalCer, need further elucidation. In this study, both β-linked glycosylceramides recognized by anti-CD1d-α-GalCer, β-ManCer and β-GalCer (C26:0), have a phytosphingosine base, whereas the one that was not recognized has a sphingosine base. The β-GalCer molecule previously reported as not being recognized anti-CD1d-α-GalCer has a 24:1 acyl chain and a sphingosine base, as well as one double bond. Based on these observations, it might be possible that β-linked glycosylceramides with a phytosphingosine base loaded to CD1d can bind to anti-CD1d-α-GalCer. Phytosphingosine is known to exist in specific tissues such as the epidermis, small intestine and kidney (41). We don't have enough comparisons to evaluate the role of the double bond in C24:1.

In contrast to synthetic preparations of β-GalCer, where the active compound could contain minute contaminations of α-anomer, β-ManCer is substantially (>20-fold) more potent than its α-linked anomer, α-ManCer. Likewise, by plasmon resonance, anti-CD1d-α-GalCer's affinity for α-ManCer is at least 60-fold lower than its affinity for β-ManCer and is difficult to detect at all. Thus, we can confidently rule out the possibility that the inhibition of activation of β-ManCer-stimulated iNKT cells by anti-CD1d-α-GalCer is due to contaminating α-ManCer rather than β-ManCer alone complexed with CD1d.

Anti-CD1d-α-GalCer's recognition of CD1d-β-ManCer complexes indicates that certain β-linked monoglycosylceramides have the capacity to assume, at least a part of the time, a structural display similar to that of CD1d-α-GalCer, which proves to be an interesting twist in our understanding of β-linked glycolipid processing and presentation to the immune system. Our results provide further evidence for the ability of anti-CD1d-α-GalCer antibodies to be important tools to monitor and even influence the biology of iNKT cell immunity.

## Data Availability Statement

All datasets generated for this study are included in the manuscript/Supplementary Files.

## Ethics Statement

The animal study was reviewed and approved by the NCI Animal Care and Use Committee.

## Author Contributions

KC, JY, JB, and MT: experimental conception and design. BC and GP: synthesis of β-ManCer. KC, JY, AB, LP, JW, DZ, and MT: development of methodology and acquisition of data. KC, JY, JW, DV, DZ, JB, and MT: analysis and interpretation of data. KC, JB, and MT: writing of the manuscript. JY, AB, JW, DV, MS, LP, BC, SC, SP, GP, and DZ: review and revision of the manuscript. JB and MT: study supervision.

### Conflict of Interest

The authors declare that the research was conducted in the absence of any commercial or financial relationships that could be construed as a potential conflict of interest.
