# Supplementary material for: Structure-Function Implications of the Ability of Monoclonal Antibodies Against α-Galactosylceramide-CD1d Complex to Recognize β-Mannosylceramide Presentation by CD1d
Source: Front Immunol. 2019 Oct 9;10:2355. doi: 10.3389/fimmu.2019.02355 (PMC6794452; doi:10.3389/fimmu.2019.02355)
Supplement: Supplementary file 1 [file Presentation_1.PPTX]

## Slide 1
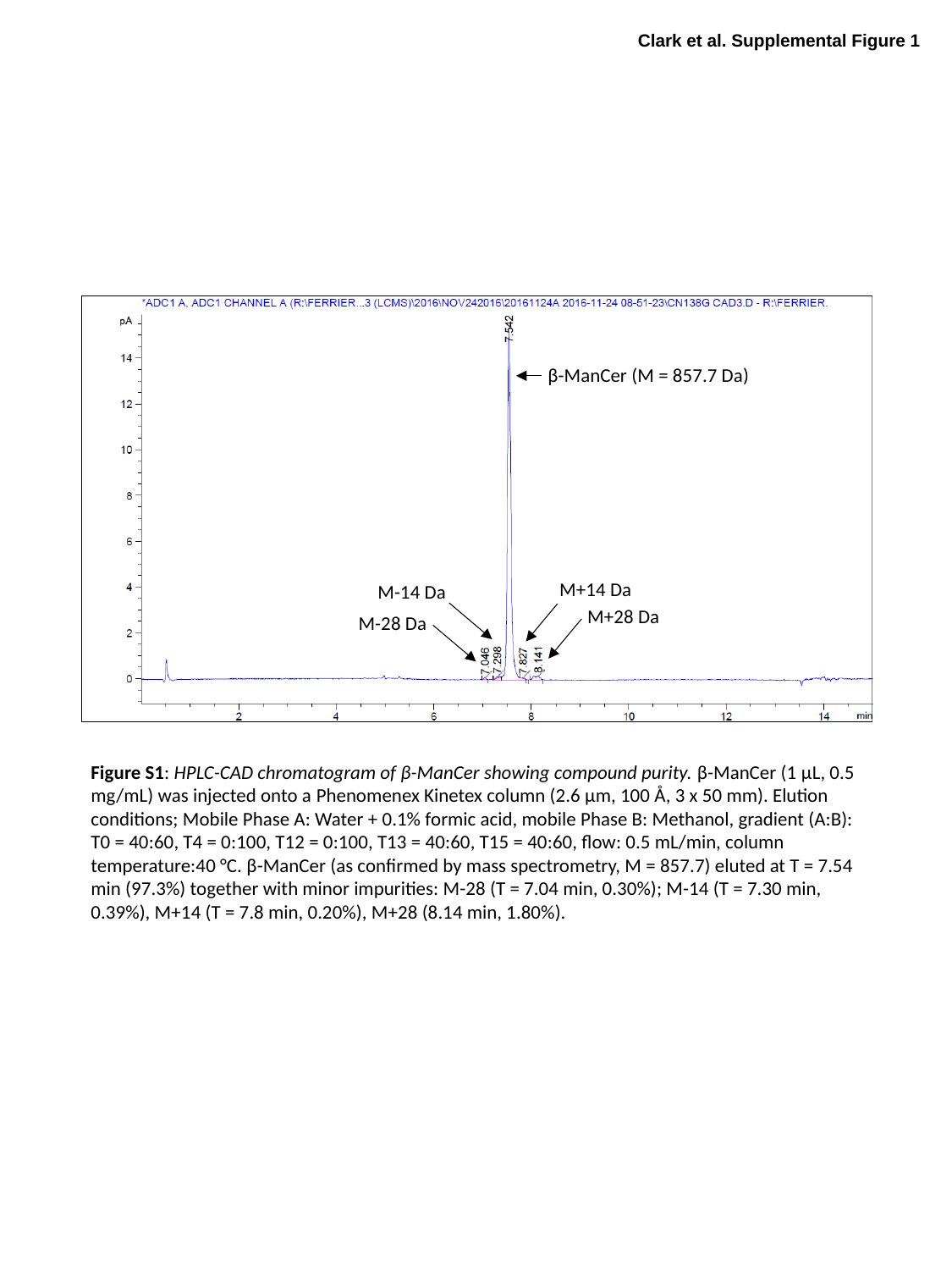

Clark et al. Supplemental Figure 1
β-ManCer (M = 857.7 Da)
M+14 Da
M-14 Da
M+28 Da
M-28 Da
Figure S1: HPLC-CAD chromatogram of β-ManCer showing compound purity. β-ManCer (1 µL, 0.5 mg/mL) was injected onto a Phenomenex Kinetex column (2.6 μm, 100 Å, 3 x 50 mm). Elution conditions; Mobile Phase A: Water + 0.1% formic acid, mobile Phase B: Methanol, gradient (A:B): T0 = 40:60, T4 = 0:100, T12 = 0:100, T13 = 40:60, T15 = 40:60, flow: 0.5 mL/min, column temperature:40 °C. β-ManCer (as confirmed by mass spectrometry, M = 857.7) eluted at T = 7.54 min (97.3%) together with minor impurities: M-28 (T = 7.04 min, 0.30%); M-14 (T = 7.30 min, 0.39%), M+14 (T = 7.8 min, 0.20%), M+28 (8.14 min, 1.80%).

## Slide 2
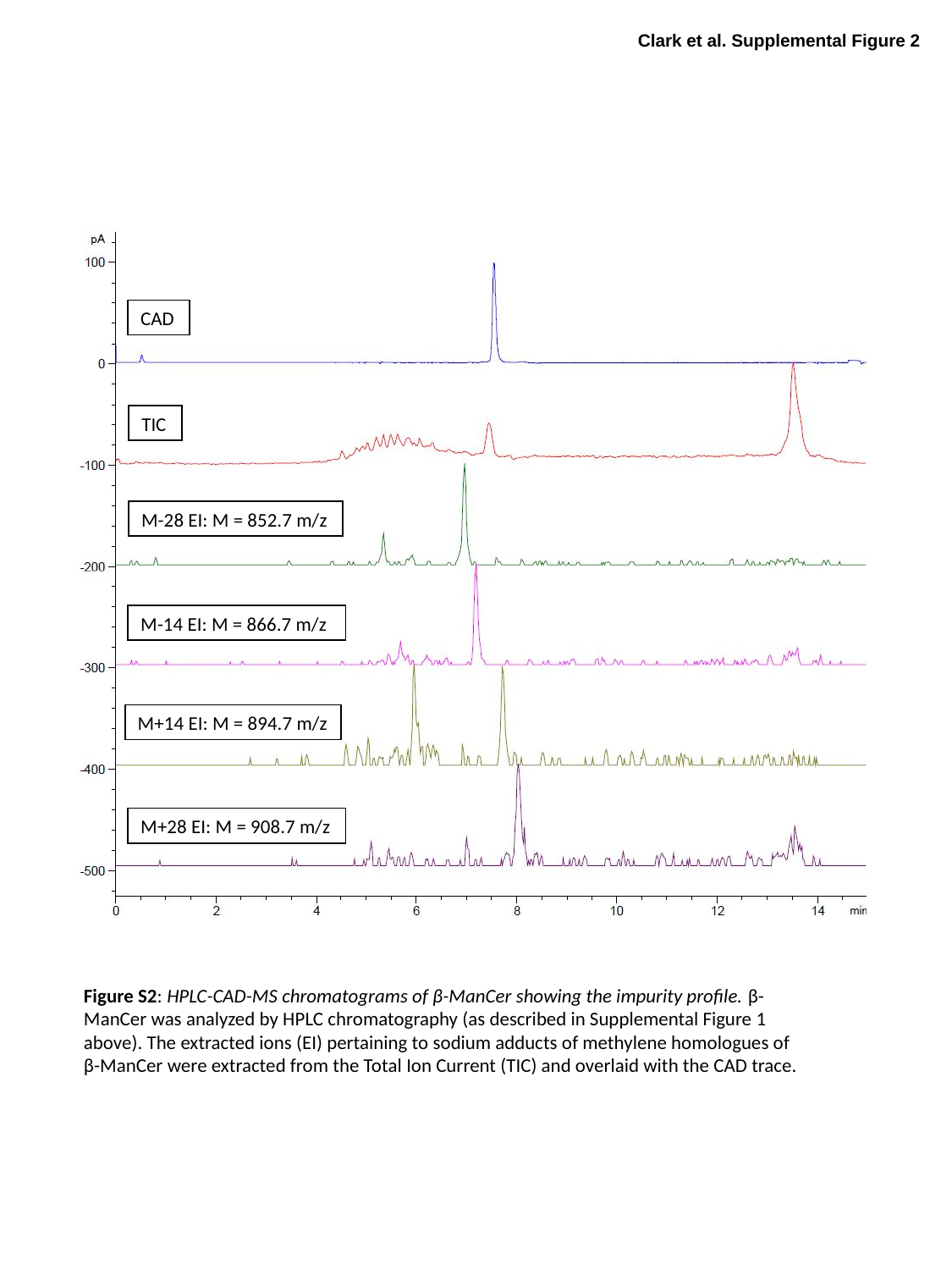

Clark et al. Supplemental Figure 2
CAD
TIC
M-28 EI: M = 852.7 m/z
M-14 EI: M = 866.7 m/z
M+14 EI: M = 894.7 m/z
M+28 EI: M = 908.7 m/z
Figure S2: HPLC-CAD-MS chromatograms of β-ManCer showing the impurity profile. β-ManCer was analyzed by HPLC chromatography (as described in Supplemental Figure 1 above). The extracted ions (EI) pertaining to sodium adducts of methylene homologues of β-ManCer were extracted from the Total Ion Current (TIC) and overlaid with the CAD trace.

## Slide 3
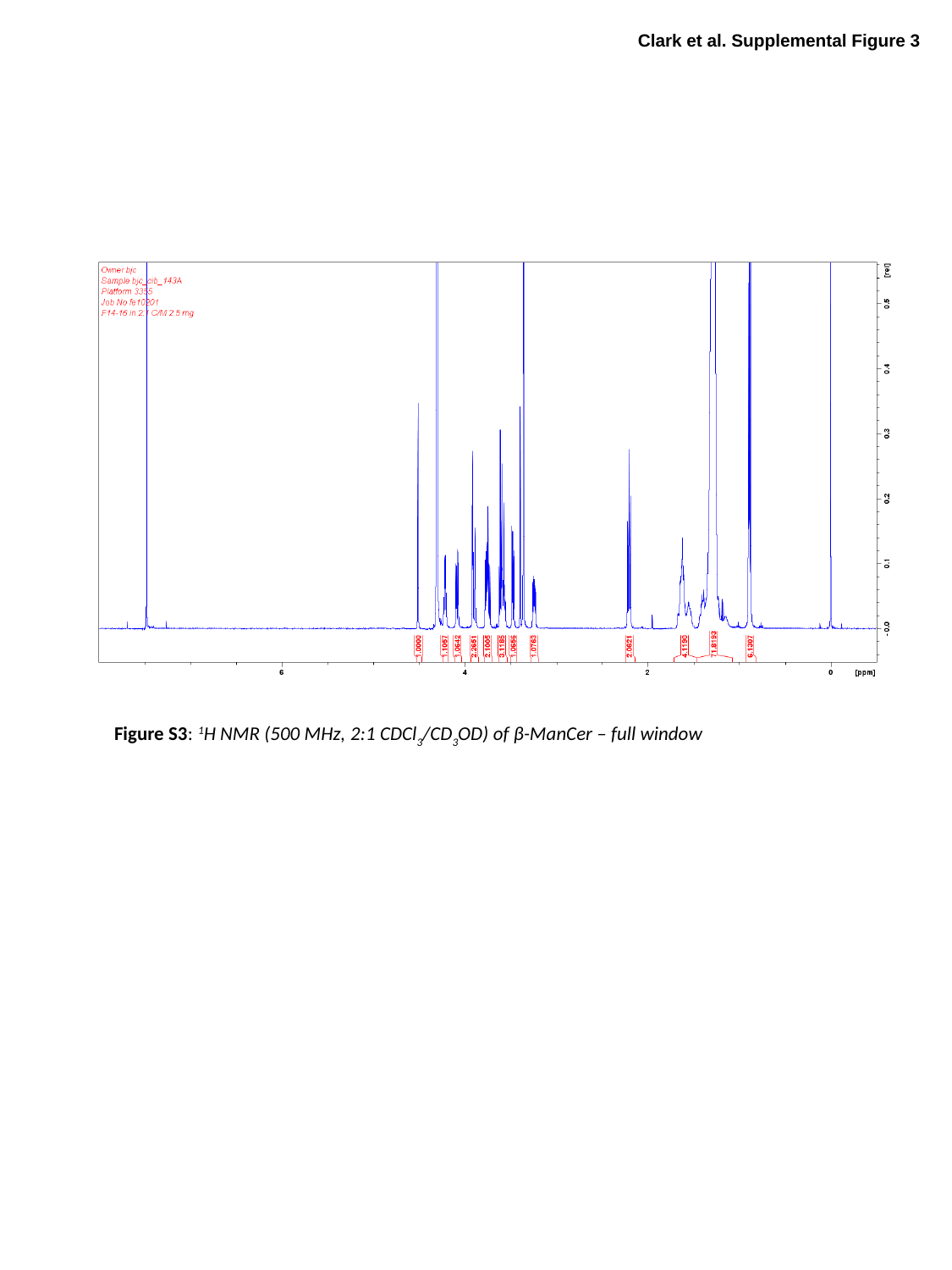

Clark et al. Supplemental Figure 3
Figure S3: 1H NMR (500 MHz, 2:1 CDCl3/CD3OD) of β-ManCer – full window

## Slide 4
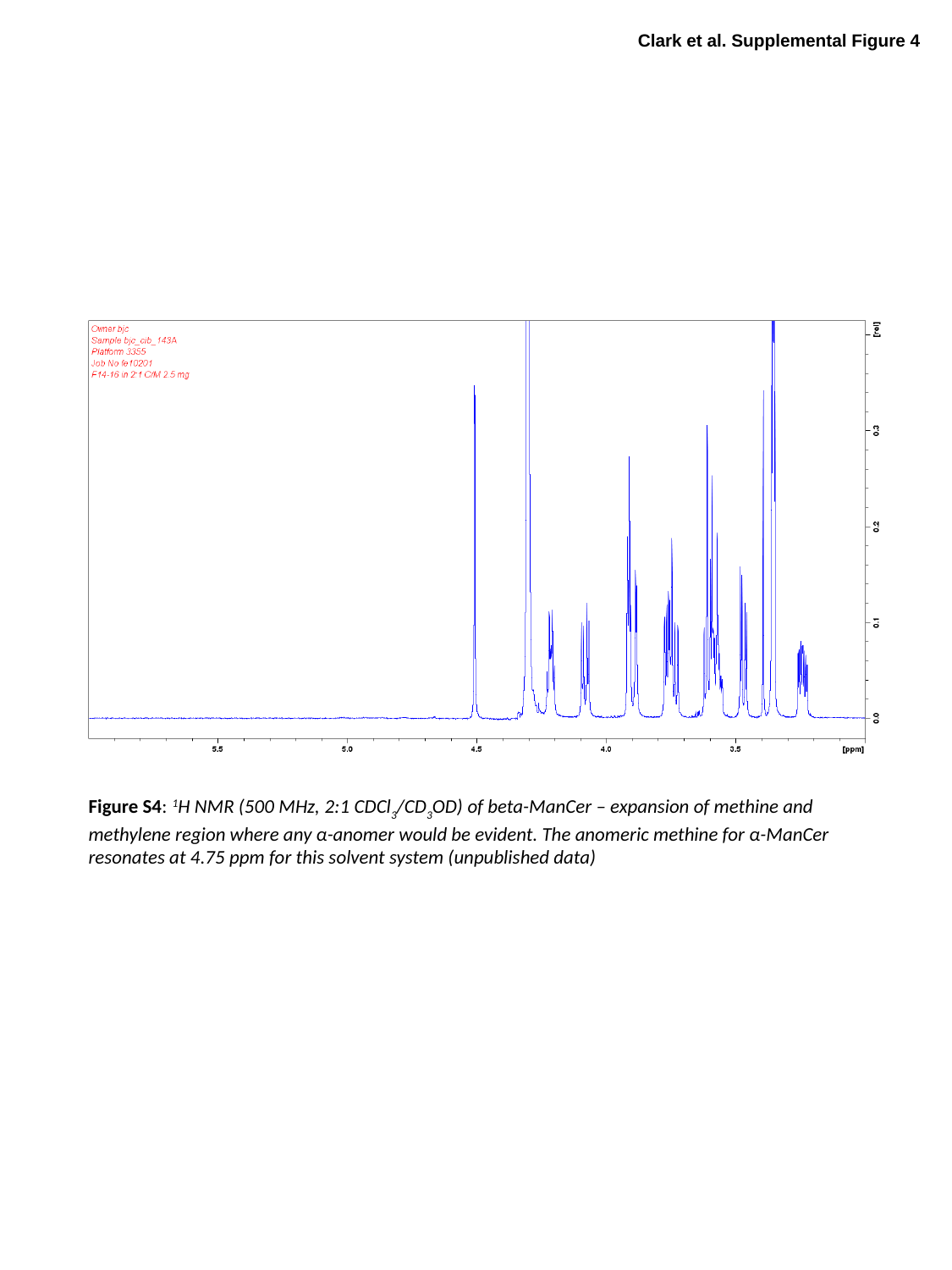

Clark et al. Supplemental Figure 4
Figure S4: 1H NMR (500 MHz, 2:1 CDCl3/CD3OD) of beta-ManCer – expansion of methine and methylene region where any α-anomer would be evident. The anomeric methine for α-ManCer resonates at 4.75 ppm for this solvent system (unpublished data)

## Slide 5
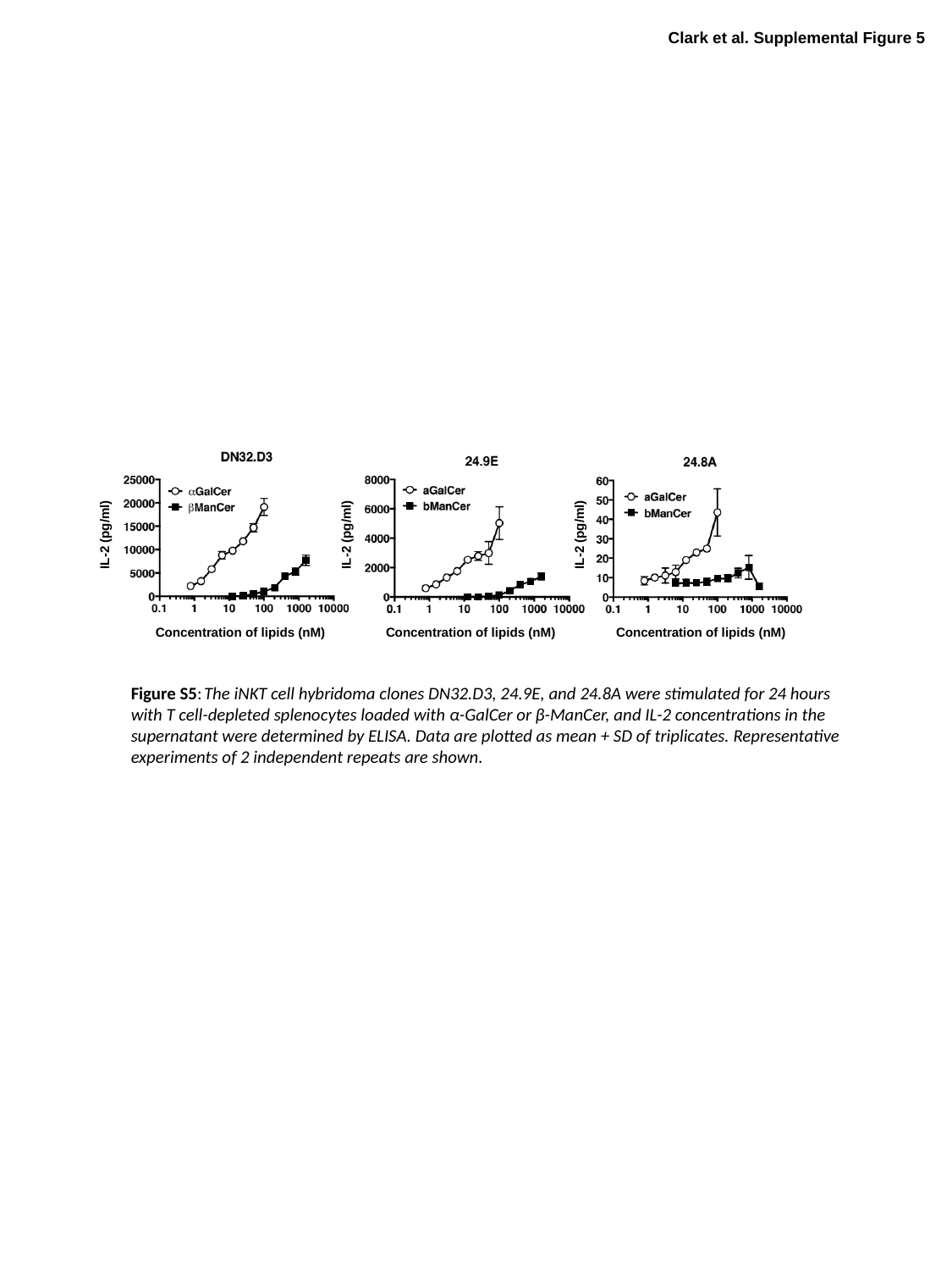

Clark et al. Supplemental Figure 5
IL-2 (pg/ml)
IL-2 (pg/ml)
IL-2 (pg/ml)
Concentration of lipids (nM)
Concentration of lipids (nM)
Concentration of lipids (nM)
Figure S5: The iNKT cell hybridoma clones DN32.D3, 24.9E, and 24.8A were stimulated for 24 hours with T cell-depleted splenocytes loaded with α-GalCer or β-ManCer, and IL-2 concentrations in the supernatant were determined by ELISA. Data are plotted as mean + SD of triplicates. Representative experiments of 2 independent repeats are shown.
